# Tunable Twin Matching Frequency (*f*_*m1*_/*f*_*m2*_) Behavior of Ni_1−x_Zn_x_Fe_2_O_4_/NBR Composites over 2–12.4 GHz: A Strategic Material System for Stealth Applications

**DOI:** 10.1038/srep44457

**Published:** 2017-03-15

**Authors:** Lokesh Saini, Manoj Kumar Patra, Raj Kumar Jani, Goutam Kumar Gupta, Ambesh Dixit, Sampat Raj Vadera

**Affiliations:** 1 Camouglage Division, Defence Laboratory, Ratanada Palace, Jodhpur-342011, India; 2 Department of Physics & Center for Solar Energy, Indian Institute of Technology Jodhpur, Old Residency Road, Jodhpur-342011, India

## Abstract

The gel to carbonate precipitate route has been used for the synthesis of Ni_1−x_Zn_x_Fe_2_O_4_ (x = 0, 0.25, 0.5 and 0.75) bulk inverse spinel ferrite powder samples. The optimal zinc (50%) substitution has shown the maximum saturation magnetic moment and resulted into the maximum magnetic loss tangent (tanδ_m_) > −1.2 over the entire 2–10 GHz frequency range with an optimum value ~−1.75 at 6 GHz. Ni_0.5_Zn_0.5_Fe_2_O_4_- Acrylo-Nitrile Butadiene Rubber (NBR) composite samples are prepared at different weight percentage (wt%) of ferrite loading fractions in rubber for microwave absorption evaluation. The 80 wt% loaded Ni_0.5_Zn_0.5_Fe_2_O_4_/NBR composite (FMAR80) sample has shown two reflection loss (RL) peaks at 5 and 10 GHz. Interestingly, a single peak at 10 GHz for 3.25 mm thickness, can be scaled down to 5 GHz by increasing the thickness up to 4.6 mm. The onset of such twin matching frequencies in FMAR80 composite sample is attributed to the spin resonance relaxation at ~5 GHz (*f*_*m1*_) and destructive interference at λ_m_/4 matched thickness near ~10 GHz (*f*_*m2*_) in these composite systems. These studies suggest the potential of tuning the twin frequencies in Ni_0.5_Zn_0.5_Fe_2_O_4_/NBR composite samples for possible microwave absorption applications.

Microwave absorbing materials are important in the present war scenario to reduce the Radar Cross Section (RCS) for strategic airborne objects^1^. These materials also important for applications in civil sector viz. communication interference between electronic devices, antenna background clutters etc. Various dielectric, magnetic and carbonaceous materials, such as barium titanate^2^, core-shell materials^3^, carbon black^4^, carbonyl iron^5^,^6^, carbon nanotubes (CNTs)^7^, iron oxide^8^ and ferrites^9^,^10^ etc. are being investigated as the functional filler materials for microwave absorption applications. There are continuous efforts to achieve the enhanced wide band MW absorption properties by converting the materials in different geometries viz. ferrite nanofibers^11^, porous hollow microrods^12^, nested structure^13^ etc. Further, among these, ferrite materials provide several advantages such as relatively smaller values of absorber thickness, particularly for lower frequencies in the MW region, tunability of ferromagnetic resonance (FMR)^14^, excellent environmental stability, etc. in comparison to the dielectric materials. Ni_1−x_Zn_x_Fe_2_O_4_ inverse spinel ferrite system attracted special attention because of possible tunable electrical^15^,^16^, magnetic^17^,^18^, and microwave absorption properties^19^,^20^,^21^,^22^ by modulating the magnetic interaction at magnetic ion sites. Ni_1−x_Zn_x_Fe_2_O_4_ (at x = 0) having the inverse spinel structure of (Fe^3+^)^tet^[Ni^2+^, Fe^3+^]^oct^O_4_ with cation’s antiparallel spin arrangements at tetragonal (A) and orthogonal (B) sites gives rise to the magnetic properties due to the superexchange interaction between A and B sites mediated through O^2−^ ions. The net magnetization (M_T_) in Ni-Zn ferrite system is the result of the difference of magnetization at octahedral (M_B_) and tetrahedral sites (M_A_) giving rise to the ferrimagnetism. The spin arrangement in Ni-Zn system can be altered by substituting Zn^2+^ ions, replacing Fe^+^ ^3^ ions at the tetragonal sites (Zn_x_Fe_1−x_^3+^)^tet^[Ni_1−x_^2+^, Fe_1+x_^3+^]^oct^O_4_. This substitution results in the significant change of magnetic interaction and thus magnetic properties^23^,^24^. The absorption frequencies of these ferrite materials depend on the electron spin arrangement and also on the domain spin orientations. The incident microwave radiation can be absorbed in ferrite materials by exciting spin relaxation process, which may be transferred to host lattice due to spin-lattice coupling. The spin-lattice interaction^25^ in spinel ferrites, which strongly affects MW absorption characteristics, depends on the distribution of cations in the crystal structure, which is not only decided by the chemical composition and synthetic route but also by the other parameters including pH of chemical reactions^26^, annealing temperature, the rate of heating/cooling, etc.

The ferrite materials need to be dispersed in suitable binder resin matrix to realize the practical microwave absorbing products. Therefore, studies on MW absorbing composites with dispersed magnetic ferrite materials in polymer/resin matrix has always been a subject of interest^27^. Dispersion of ferrite granules has been attempted in different host matrices viz. epoxy resin^28^,^29^, polyvinylchloride (PVC)^30^, wax^31^,^32^ etc., wherein the studies have been focused on understanding the variation of electromagnetic characteristics of composites with a loading percentage of filler materials as well as their dispersion behavior. Further, the filler-host matrix interactions facilitate multiple scattering of MW radiation via suspended filler granules which may significantly enhance the MW absorption properties in the composites^33^. Yet, there are numerous challenges in the synthesis of homogeneous composite system e.g. dispersion/suspension of the high-density ferrite powder in liquid resin/wax medium leading to the inhomogeneities in the composites. The elastomeric matrix may provide a relatively better host for uniform dispersion of high-density filler due to the entrapment of heavy ferrite granules in cross-linked rubber structure during mixing and vulcanization process^34^. In spite of these advantages, there are very few studies on ferrite-elastomeric composites, directing to microwave absorption. Considering these advantages, in this report, we have focused our studies on the microwave absorbing properties of Ni_1−x_Zn_x_Fe_2_O_4_ ferrite as filler in Acrylo-Nitrile Butadiene Rubber (NBR) elastomeric matrix due to its light weight, flexible nature, high elongation, resistance to the corrosion, good high/low temperature stability (−20 °C to +125 °C) and high values of tensile strength. Ni_1−x_Zn_x_Fe_2_O_4_ (x = 0, 0.25, 0.50 and 0.75) ferrite powders have been prepared using simple, scalable and energy efficient wet chemical method. As synthesized powders have been annealed at different temperatures to study their size-dependent physical and microwave absorption properties. Flexible sheets were fabricated in NBR host matrix with different loadings fractions in the range of 50–80 weight percent (wt%) of the Ni-Zn ferrite material with optimal Zn concentration showing maximum saturation magnetization and investigated for their reflection loss characteristics in the microwave frequency range of 2–12.4 GHz.

## Results and Discussion

**Figure 1:**
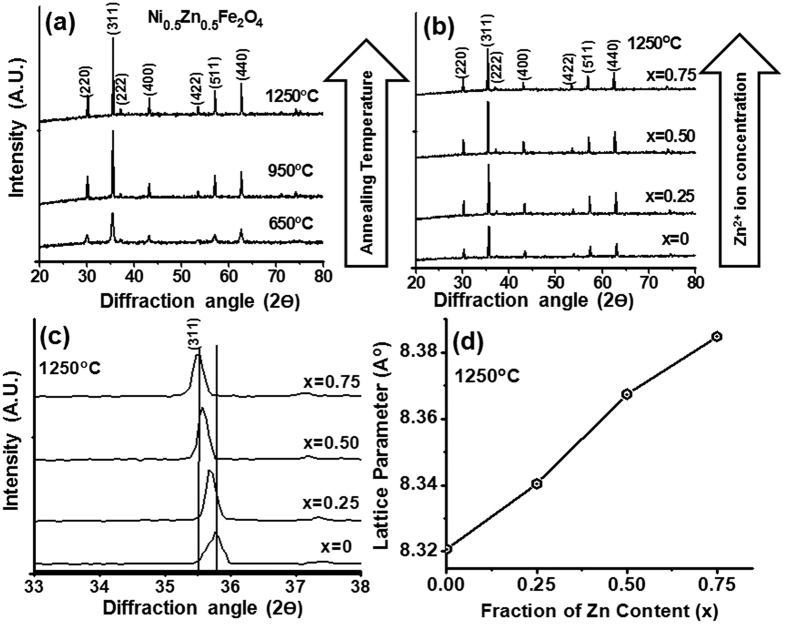
(**a**) XRD pattern of Ni_0.5_Zn_0.5_Fe_2_O_4_ ferrite powders annealed at different elevated temperatures of 650 °C, 950 °C and 1250 °C. (**b**) XRD pattern of Ni_0.5_Zn_0.5_Fe_2_O_4_ ferrite powders with Zn^2+^ ion concentration (**c**) Shifting in (311) plane XRD peak with Zn^2+^ ion concentration (**d**) Variation in lattice parameter with Zn content in compositions.

The representative XRD spectra are shown in Fig. 1a for Ni_1−x_Zn_x_Fe_2_O_4_ (x = 0.50) ferrite powder sample, annealed at 650 °C, 950 °C and 1250 °C temperatures. International Centre for Diffraction Data (ICDD) powder diffraction file (PDF) # 22–106 has been used as a reference for phase identification and all the observed diffraction peaks could be indexed with this reference. The appearance of characteristics spinel phase peaks due to (220), (311), (222), (400), (422), (511) and (440) planes, marked in Fig. 1a, confirm the formation of pure spinel phase of Ni_0.5_Zn_0.5_Fe_2_O_4_ annealed at 650 °C. The broadening of XRD peaks for 650 °C annealed powder suggests the formation of ultrafine particles. Further, enhancement of intensities and decrease of respective broadening for these diffraction peaks have been observed with the increase in sequential annealing temperatures at 950 °C and 1250 °C, respectively. The other compositions of Ni_1−x_Zn_x_Fe_2_O_4_ (x = 0, 0.25 and 0.75) ferrite powders showed similar XRD spectra (Fig. 1b). However, the diffraction peaks are relatively shifted ~0.26° in 2ϴ towards lower angle with an increase in Zn^2+^ ion concentration from NiFe_2_O_4_ to Ni_0.25_Zn_0.75_Fe_2_O_4_ as shown in Fig. 1c, attributed to the enlargement of cubic lattice parameter ‘*a*’ for these inverse spinel compounds from 8.321A° for pure Ni ferrite (x = 0) to 8.385A° for Ni_0.25_Zn_0.75_Fe_2_O_4_ferrite (x = 0.75) composition, as shown in Fig. 1d. The increased ‘*a*’ with Zn^2+^ ion concentration is caused by the replacement of Ni^2+^ ion (ionic radii ~0.78A°) to the large sized Zn^2+^ ion (ionic radii ~0.83A°) in the crystal lattice of cubic NiFe_2_O_4_ structure^35^,^36^,^37^.

**Figure 2:**
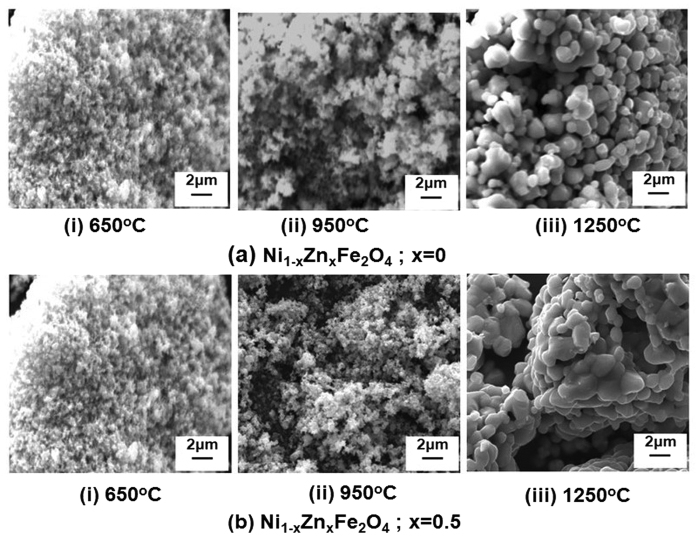
SEM micrograph of Ni_1−x_Zn_x_Fe_2_O_4_ powder annealed at elevated temperatures of 650 °C, 950 °C and 1250 °C for (**a**) x = 0 (**b**) x = 0.5.

Scanning electron microscope (SEM) micrographs are shown in Fig. 2a,b, for Ni_1−x_Zn_x_Fe_2_O_4_ powders (x = 0 and 0.5), annealed at 650 °C, 950 °C and 1250 °C. Ferrite powder samples, annealed at 650 °C, show fine microstructure with spherical particle morphology, Fig. 2a(i). The grain growth has been observed with annealing temperature, resulted into the distorted spherical morphology observed for the powder samples, annealed at 1250 °C, Fig. 2a(iii). Ni_1−x_Zn_x_Fe_2_O_4_ (x = 0.50) showed larger grain size with fused particles, as shown in Fig. 2b (iii). This observation indicates that the addition of Zn promotes the grain growth in Ni_1−x_Zn_x_Fe_2_O_4_ ferrite composition. The results are consistent with the reported literature^38^.

**Figure 3:**
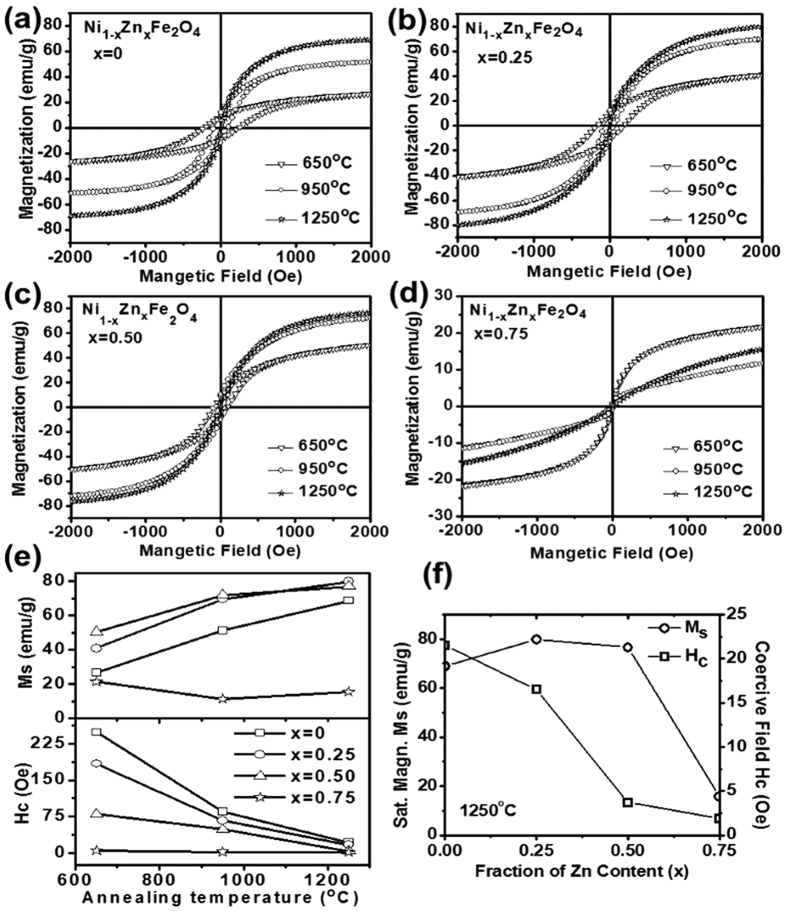
Room-temperature M-H Hysteresis curve of Ni_1−x_Zn_x_Fe_2_O_4_ ferrite powders at (**a**) x = 0 (**b**) x = 0.25 (**c**) x = 0.50 (**d**) x = 0.75 (**e**) variation of M_s_ with annealing temperature for each composition of ferrite powders (**f**) variation of saturation magnetization M_s_ and coercive field H_c_ with Zn concentration.

The room temperature field dependent magnetic measurements are summarized in Fig. 3a–d for Ni_1−x_Zn_x_Fe_2_O_4_ (x = 0, 0.25, 0.50 and 0.75) ferrite powders annealed at 650 °C, 950 °C and 1250 °C. All these compositions show ferrimagnetic behavior with different saturation magnetization (M_s_) and coercive field (H_c_) values. The variation of M_s_ and H_c_ with respect to annealing temperature and Zn^2+^ ion concentration and composition are plotted in Fig. 3e and f respectively. The saturation magnetization increases with increasing the annealing temperature for all these compositions except for the Ni_0.25_Zn_0.75_Fe_2_O_4_ ferrite powder. The marginal decrease in saturation magnetization has been observed for this composition (x = 0.75) while annealed at 950 °C and then a small increase in value for annealing at a higher temperature ~1250 °C. Similarly, the coercive field values were found to decrease with increase in annealing temperature for all the compositions except for ferrite powder with high Zn content (x = 0.75). The distribution of ions in tetrahedral and octahedral sites takes place during the thermal treatment for the formation of inverse spinel crystallographic phase. The cation ordering increases with increasing the temperature, resulting in the higher saturation magnetization, as observed in samples annealed at 1250 °C^25^,^39^. Further, saturation magnetization increases initially with an increase in Zn^2+^ ion concentration and a significant decrease in value has been observed beyond x = 0.5 for ferrite composition as shown in Fig. 3f. At low concentration of Zn^2+^ ions, spin site occupancy reduces at A (tetrahedral) sites due to the presence of non-magnetic Zn^2+^ ions, however, net magnetization increases due to the shift of Fe^3+^ ions from A site to B (octahedral) sites. The increase in Zn^2+^ substitution beyond x = 0.5 resulted in the reduced effective spin A site contributions due to the non-availability of the minimum number of spins sites for the oxygen mediated super-exchange interaction between A and B cation sites, leading to decrease in magnetization value, as observed in the present study^17^,^37^. Moreover, the annealing temperature has favored grain growth, resulting in the enhanced number of domain walls or even larger domains within the grains. The contribution of a domain wall in magnetization/demagnetization process dominates against domain rotation, as domain wall movement is less energetic than the domain wall rotation^18^. Therefore, such energy competition between domain rotation and domain wall movement in larger grains has led to the lower coercive field values for compositions annealed at high temperature i.e. 1250 °C. The decrease in coercive field values is attributed to the decrease in magneto-crystalline anisotropy with Zn substitution, as substantiated in our experimental observations^40^.

**Figure 4:**
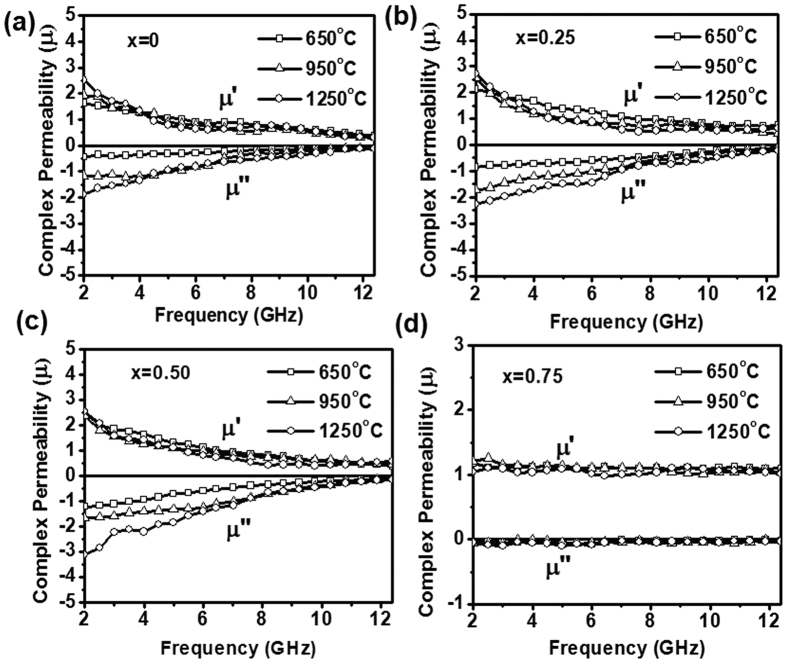
Frequency dependence of the complex permeability for annealed Ni_1−x_Zn_x_Fe_2_O_4_ powder (**a**) x = 0 (**b**) x = 0.25 (**c**) x = 0.50 (**d**) x = 0.75.

**Figure 5:**
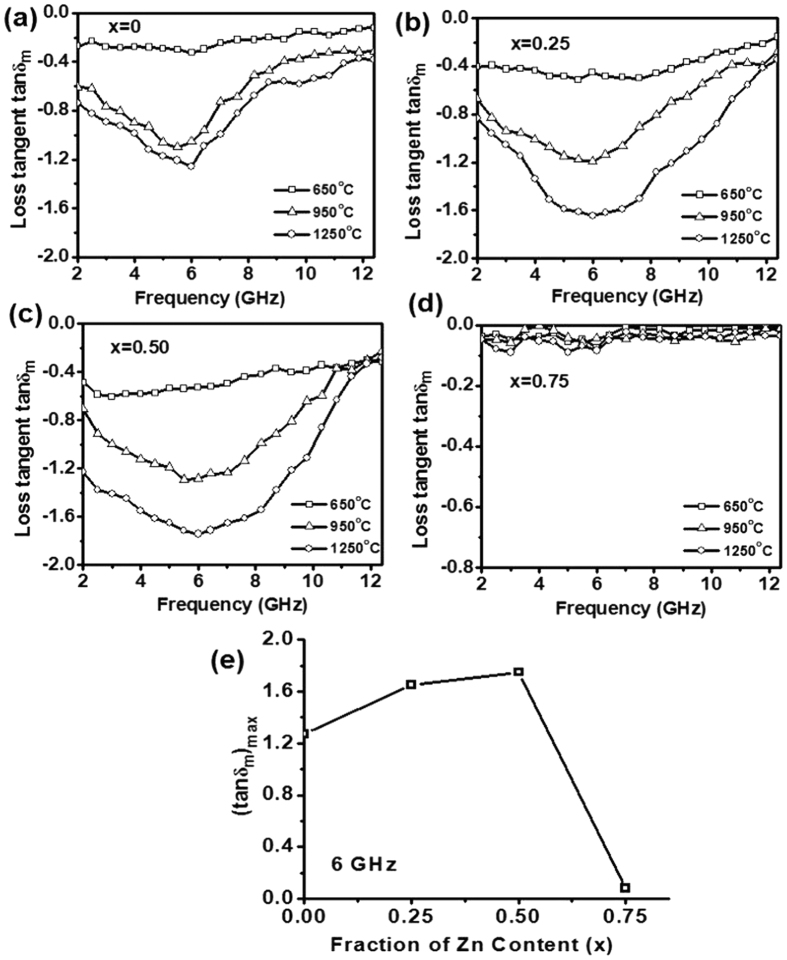
Frequency dependence of the loss tangent (tanδ_m_) for annealed Ni_1−x_Zn_x_Fe_2_O_4_ powder (**a**) x = 0 (**b**) x = 0.25 (**c**) x = 0.50 (**d**) x = 0.75 (**e**) variation of maximum loss tangent with Zn concentration at resonating frequency of 6 GHz.

The complex permittivity (ε_r_* = ε_r_′ − jε_r_″) and permeability (μ_r_* = μ_r_′ − jμ_r_″) values were computed by Nicolson and Ross algorithm using S-parameters measured through waveguide transmission line technique over MW frequency range (2–12.4 GHz)^41^. The imaginary values of complex EM parameters (ε_r_″, μ_r_″) represent the MW attenuation/absorption characteristics of material and are depicted on the negative scale, following the standard convention for representing the loss factor. All the Ni_1−x_Zn_x_Fe_2_O_4_ samples annealed at 1250 °C show similar values of ε_r_′~6.3 ± 0.1 over the measured frequency range 2–12.4 GHz. The imaginary permittivity values exhibit non-dispersive behavior with a constant value nearly ~0.005 for all Ni_1−x_Zn_x_Fe_2_O_4_ samples over the entire frequency range. These measurements suggest that Ni_1−x_Zn_x_Fe_2_O_4_ samples do not show any dielectric loss tanδ_e_ (=ε_r_″/ε_r_′) over the frequency range 2–12.4 GHz due to negligible imaginary permittivity. The measured real relative permeability (μ_r_′) values are decreasing from ~2.5 to ~1 for Ni_1−x_Zn_x_Fe_2_O_4_ (x = 0, 0.25, 0.50 and 0.75) ferrite compositions annealed at different temperatures over the 2–12.4 GHz frequency range, as shown in Fig. 4a–d. The imaginary relative permeability (μ_r_″) values also change from ~−3.12 to −0.11 simultaneously with an increase in annealing temperature for Ni_1−x_Zn_x_Fe_2_O_4_ (x = 0, 0.25 and 0.50) ferrite compositions with a dispersive behavior as shown in Fig. 4a–c for the 2–12.4 GHz frequency range. Further, μ_r_″ values increase relatively in the 2–8 GHz low-frequency region and the effect is more prominent for Ni_0.5_Zn_0.5_Fe_2_O_4_. The values are negligible ~−0.02 for all different temperature annealed Ni_0.25_Zn_0.75_Fe_2_O_4_ samples, as shown in Fig. 4d. These measurements suggest that both annealing temperature and Zn^2+^ ion concentration in Ni_1−x_Zn_x_Fe_2_O_4_, affect μ_r_″ dispersive behavior over the frequency range 2–12.4 GHz. The imaginary permeability, which is responsible for MW energy loss, depends on saturation magnetization (M_s_) and coercive field (H_c_) as given in equation (1)^42^


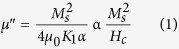


where M_s_ is saturation magnetization, K_1_ is anisotropic constant, α is Gilbert constant, H_c_ is a coercive field and μ_0_ is intrinsic permeability. The μ_r_″ values show an increasing trend from −1.90 to −3.2 at 2 GHz with an increase in annealing temperature from 650 °C to 1250 °C. This is in accordance with the observed increase in saturation magnetization M_s_ and the decrease in coercive field H_c_ for the samples (x = 0 to x = 0.5). The magnetic loss tangent (tanδ_m_ = μ_r_″/μ_r_′) for Ni_1−x_Zn_x_Fe_2_O_4_ (x = 0, 0.25, 0.50 and 0.75) ferrite powder samples, annealed at different temperatures viz. 650 °C, 950 °C and 1250 °C, are plotted in Fig. 5a–d. An increase in the loss tangent values has been observed for Ni_1−x_Zn_x_Fe_2_O_4_ ferrite samples for both (i) increase in x from 0 to 0.5 and (ii) annealing temperature from 650 °C to 1250 °C. The 1250 °C annealed Ni_1−x_Zn_x_Fe_2_O_4_ sample showed tanδ_m_ > −0.8 for x = 0 and 0.25 over the 2–8 GHz and 2–10 GHz frequency bands with the maximum tanδ_m_ ~−1.27 (x = 0) and ~−1.65 (x = 0.25) at ~6 GHz frequency. The sample with x = 0.5 has shown even enhanced tanδ_m_ > −1.2 over 2–10 GHz with maximum value ~−1.75 at 6 GHz. These measurements suggest that the maxima of loss tangent (tanδ_m_)_max_ values increase with Zn^2+^ ion concentration up to x = 0.50 and beyond that at x = 0.75 reduced sharply to the negligible loss tangent ~−0.05 as shown in Fig. 5e. These observations are consistent with the behavior of saturation magnetization and coercive field values as described earlier in the text. Further, it is interesting to note that the (tanδ_m_)_max_ for all compositions (x = 0, 0.25, 0.50 and 0.75) is found at 6 GHz, nearly independent of the frequency. The Ni_0.5_Zn_0.5_Fe_2_O_4_ ferrite powder annealed at 1250 °C shows maximum loss tangent values among all other compositions and therefore, has been considered for preparation of rubber composites for microwave absorption applications.

**Figure 6:**
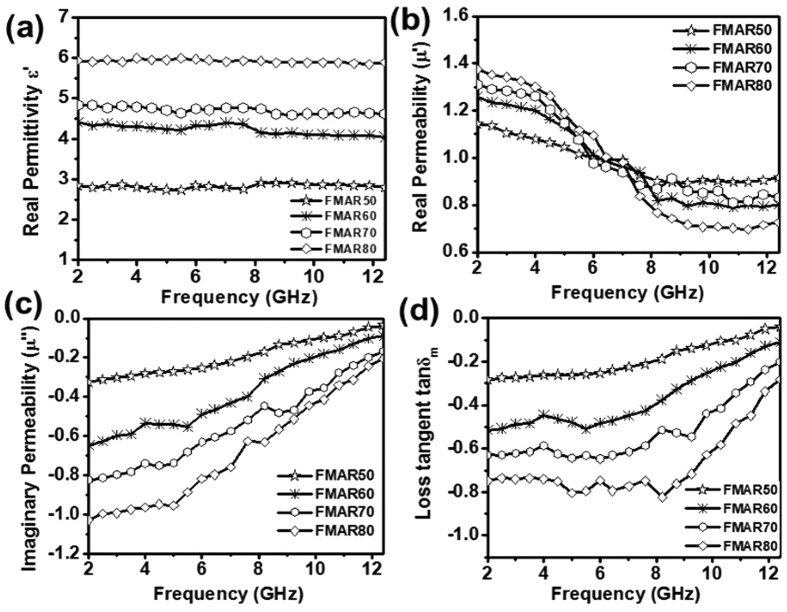
(**a**) Real permittivity plots for ferrite loaded rubber composites FMAR50-80 with MW frequencies (**b**) real permeability behavior of ferrite loaded rubber composites (**c**) imaginary permeability plots of ferrite loaded rubber composites (**d**) loss tangent profiles for ferrite loaded rubber composites.

**Figure 7:**
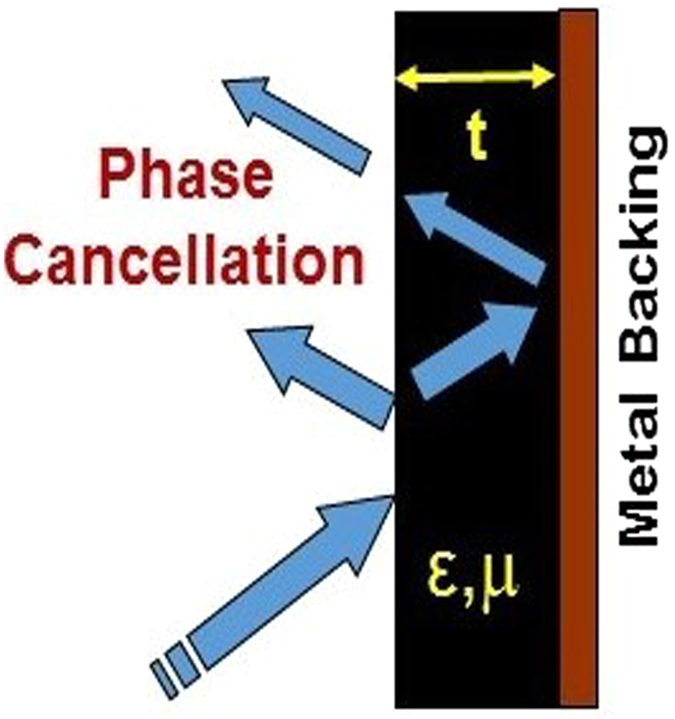
A schematic representation of reflection loss mechanism for incident MW radiation at optimal absorber thickness.

A 50 wt% loaded ferrite-NBR sample, FMAR50, showed the real permittivity (ε_r_′) ~2.8 ± 0.1 over the entire 2–12.4 GHz frequency range. With the increase in Ni_0.5_Zn_0.5_Fe_2_O_4_ ferrite filler loading, ε_r_′ value has increased to ~4.2 ± 0.2 at 60 wt% loading in sample FMAR60, ~4.7 ± 0.1 at 70 wt% in sample FMAR70 and ~5.9 at 80 wt% loading in sample FMAR80 as shown in Fig. 6a. The values of the imaginary component of the permittivity are found negligible between ~−0.0015 and ~−0.002 for all the different ferrite loaded rubber composites over the entire frequency range of 2–12.4 GHz. This small value of complex permittivity suggests the absence of dielectric loss contribution for microwave absorption in these rubber composites. The real component of the permeability values (μ_r_′) in rubber composite samples has shown a decrease with increase in frequency as shown in Fig. 6b and μ_r_′ values lie in the range of 1.14 to 0.90, 1.25 to 0.80, 1.26 to 0.83 and 1.37 to 0.70, for FMAR50, FMAR60, FMAR70, and FMAR80 samples respectively. The imaginary permeability (μ_r_″) components for FMAR50 sample is found to be in the range of ~−0.33 to −0.03 with reducing trend towards the higher frequency range. Further, enhancement of μ_r_″ values has been observed with increase in loading fraction of Ni_0.5_Zn_0.5_Fe_2_O_4_ ferrite powder in composite samples. In FMAR60 sample, the μ_r_″ shows wider dispersion characteristics with values in the range of ~−0.65 to −0.10 over the entire frequency range. The similar trend has been observed for FMAR70 and FMAR80 samples where μ_r_″ values fall in the range ~−0.82 to −0.17 and ~−1.03 to −0.20 respectively as shown in Fig. 6c. The magnetic loss tangent (tanδ_m_) values show wide dispersion and values range from ~−0.28 to −0.03, −0.50 to −0.11, ~−0.63 to −0.20 and ~−0.80 to −0.30 in the entire 2–12.4 GHz frequency range for FMAR50, FMAR60, FMAR70 and FMAR80 samples respectively, as summarized in Fig. 6d. Thus, we observed that the magnetic loss tangent values have increased with Ni_0.5_Zn_0.5_Fe_2_O_4_ loading fraction in these rubber composite samples with decreasing trend towards higher frequency range. The observed enhancement in loss tangent values has been attributed to the increasing loading fraction of ferrite in NBR by 1.50, 2.33 and 4.00 times in comparison to sample FMAR50 (Table 1). The MW reflection losses (R.L.) have been computed for Ni_0.5_Zn_0.5_Fe_2_O_4_ ferrite loaded composite samples (FMAR50 - FMAR80) by using the following equation (2)^43^:


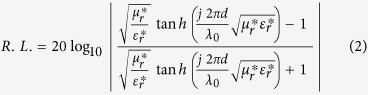


where, λ is the wavelength, μ_r_^*^ is the complex relative permeability, ε_r_^*^ is the complex relative permittivity and *d* is the absorber thickness. Further, the impedance of absorber’s top layer should be comparable to the free space impedance Z_0_ (377 Ω) to enter the microwaves inside the absorber for its effective attenuation as explained schematically in Fig. 7. Considering the close approximation of matched wave impedance criterion, the reflection loss of absorber should have matched values for absorber thickness (*d*_*m*_) at *λ*_*m*_/4, to satisfy the destructive interference criterion among surface reflected and metal back-reflected wave with the following dependence, as explained by eqs (3) and (4) (Fig. 7)^32^,^44^


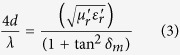



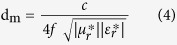


**Figure 8:**
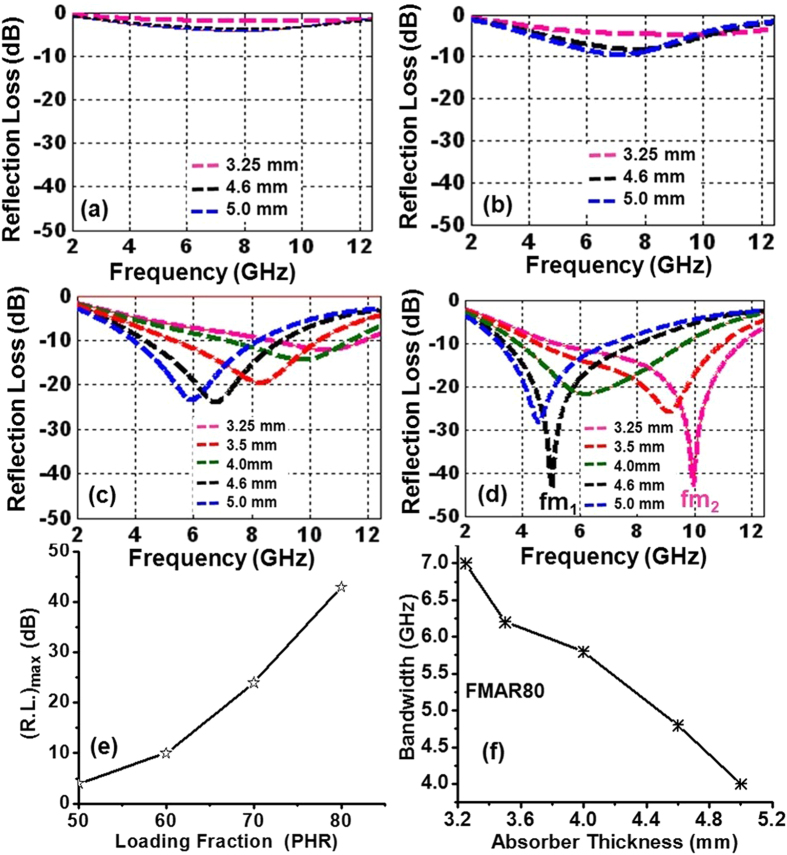
Optimal Reflection Loss (R.L.) over frequency range 2–12.4 GHz and matching thicknesses of ferrite loaded rubber composites (**a**) FMAR50 (**b**) FMAR60 (**c**) FMAR70 (**d**) FMAR80 (**e**) variation of maximum reflection loss with ferrite powder loading fraction in composite (**f**) optimal R.L. bandwidth profile with composite absorber thickness.

The calculated reflection loss values for different Ni_0.5_Zn_0.5_Fe_2_O_4_ ferrite loaded samples are shown in Fig. 8a–d. FMAR50 sample shows the maximum possible reflection loss value (R.L.)_max_ ~−4dB at ~8 GHz for 5 mm absorber thickness, which has increased up to ~−10 dB at ~7 GHz for FMAR60 sample for the same thickness. Similarly, with further increase of ferrite loading (FMAR70), the (R.L.)_max_ value has further increased to ~−15 dB at 10 GHz, ~20 dB at 8.3 GHz and ~24 dB at 7 GHz for absorber thickness values of ~3.5 mm, 4.0 mm, and 4.6 mm, respectively. It is interesting to note that the absorption peak is shifting towards the lower frequency with increasing the absorber thickness for this composition. The observed shifting in reflection loss, towards lower frequency for FMAR70 composite can be explained using eqs (3 and 4). These relations suggest that absorber thickness will effectively reduce with increasing ε_r_′ and tanδ_m_. The insignificant values of reflection loss observed for FMAR50 and FMAR60 are mainly because of the relatively lower tanδ_m_, for these composite samples as shown in Fig. 6d. Interestingly, the onset of twin match frequencies (*f*_*m1*_/*f*_*m2*_) has been observed at two different matched thicknesses (*t*_*m1*_ and *t*_*m2*_), Fig. 8d, for FMAR80 composite sample. At absorber thickness *t*_*m1*_ = 4.6 mm, the first matched (R.L.)_max_ value is ~−43 dB at the first matching frequency *f*_*m1*_~5 GHz. With the decrease in absorber thickness, R.L profile has shifted towards higher frequency side with lower (R.L.)_max_ values ~−22 dB and ~−26 dB for absorber thicknesses ~4 mm and 3.5 mm, respectively. On further reduction in the absorber thickness at *t*_*m2*_ = 3.25 mm, the second matched (R.L.)_max_ value ~−43 dB has been observed at the second matching frequency *f*_*m2*_ ~10 GHz.

The existence of such two matching frequencies has been observed earlier in pure ferrite powders, however, in rubber-ferrite composite samples only a single matching frequency has been reported^37^,^45^. The two possible MW absorption loss mechanism are generally attributed in ferrite materials: (i) relaxation mechanism due to the domain wall motion and (ii) the spin rotational resonance. The domain wall resonance mechanism usually dominates at lower MW frequency range (<1 GHz), as the relaxation of domain motion does not follow the higher MW frequency beyond 1 GHz, whereas relaxation of spin resonance or ferromagnetic resonance (FMR) dominates at the higher MW frequencies. The maximum loss tangent values observed at ~6 GHz for 1250 °C annealed Ni_0.5_Zn_0.5_Fe_2_O_4_ ferrites, is close to the first matching frequency (*f*_*m1*_) observed at ~5 GHz for 4.6 mm thick FMAR80 sample as shown in Fig. 8d, and is attributed to the spin resonance relaxation mechanism in these samples^46^. As discussed earlier, the FMR frequencies and thus, MW absorption of Ni-Zn ferrite system depends strongly on chemical composition, synthetic route and post processing conditions. Shi *et al*. tuned the resonance frequency ~2.54 GHz for Ni spinel ferrite nanoparticles prepared using sol-gel process^47^ whereas Zhao *et al*. adjusted the matching absorption frequencies for Ni-Zn spinel ferrite in the range of ~1–12 GHz by substitution of Cu^2+^/Co^2+^ ions^48^. The second matching frequency (*f*_*m2*_) has been observed at ~10 GHz for 3.25 mm FMAR80 absorber thickness. The *f*_*m2*_ is independent of resonance frequency and is observed because of matched absorber thickness (λ_m_/4 ~ 3.25 mm) as given by eq. (4). The R.L. profile associated to *f*_*m1*_ at absorber thickness ~4.6 mm may extend towards the higher frequency side with a decrease in thickness up to 3.5 mm. Whereas, the R.L. profile associated with *f*_*m2*_ may contribute towards lower frequency side with an increase in thickness up to 4 mm. It has been observed that R.L. profiles have both the components of matching frequencies (*x.f*_*m1*_ + *y.f*_*m2*_) at these thicknesses, where *x* and *y* are the fractional contribution from individual matching frequencies *f*_*m1*_ and *f*_*m2*_, respectively. At the intermediate absorber thickness of 3.5 mm and 4.0 mm the matching frequency (*f*_*M*_) behavior follows the trend as given in eqs (5 and 6)


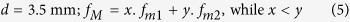



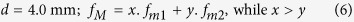


The matching frequencies (*f_m_*) for *d* = 3.5 mm and 4.0 mm thick FMAR80 samples are 9 and 6 GHz respectively, as shown in Fig. 8d. The fractional contributions (*x* and *y*) for *d* = 3.5 mm thick FMAR80 sample are *x* = 0.2 and *y* = 0.8 corresponding to *f*_*m1*_ (5 GHz) and *f*_*m2*_ (10 GHz) respectively. For *d* = 4.0 mm thick FMAR80 sample, the fractional contributions are *x* = 0.8 and *y* = 0.2, corresponding to *f*_*m1*_ (5 GHz) and *f*_*m2*_ (10 GHz) respectively, substantiating the proposed mechanism of twin matching frequencies contribution for microwave absorption in these ferrite-rubber composite samples.

## Conclusions

The maximum reflection loss (R.L.)_max_ values increase with loading fraction of Ni_0.5_Zn_0.5_Fe_2_O_4_ ferrite powder in these rubber composites, as shown in Fig. 8e. Interestingly, for FMAR80 the desired frequency bandwidth (where R.L. ≥ 10 dB or at least 90% attenuation of MW signal) decreases with absorber thickness as shown in Fig. 8f. The MW absorber at 3.25 mm thickness (corresponding to *f*_*m2*_) has the widest bandwidth of ~7 GHz, whereas, at absorber thickness of 4.6 mm, frequency bandwidth window reduced to ~4.2 GHz. In the intermediate thicknesses, the bandwidth lies between this ranges. These studies suggest that the FMAR80 absorber can be used as a tunable MW frequency absorber using the thickness as a controlling parameter for strategic applications such as microwave stealth over the different frequency bands.

## Methods

### Experimental details

The ferrite powders are prepared using gel to carbonate precipitation method, where the co-precipitations of divalent cations (Ni^2+^, Zn^2+^) as fine particles of carbonates within hydrated gels of ferric hydroxide has been carried out by adding the alkali solution into the mixed metal salt ions. 1 M solution of FeCl_3_ (Merck CAS No. 7705-08-0), NiCl_2_ (Merck CAS No. 7718-54-9), ZnCl_2_ (Merck CAS No. 7646-85-7) were mixed stoichiometrically, followed by adding dropwise 1 M Na_2_CO_3_ solution (Merck CAS No. 497-19-8) while maintaining pH ~9, to realize the co-precipitation. The obtained precipitate was washed and dried in an oven at ~90 °C for 2 hours in the air ambient. The resultant composite powder consists of sub-micron crystalline particles of carbonates, embedded within the amorphous medium of Fe_2_O_3_.xH_2_O (70< x <110). The composite powder was annealed sequentially at 650 °C, 950 °C and 1250 °C for 3 hours each to obtain final sample, which was used for detailed characterization of the physical and microwave absorption properties. The similar process has been followed to prepare a series of Ni_1−x_Zn_x_Fe_2_O_4_ spinel ferrites with x = 0, 0.25, 0.50 and 0.75, to understand the effect of Zn substitution on the physical and microwave absorption properties.

Rubber based microwave absorbing sheets are fabricated using mechanical milling followed by high-temperature compression moulding (HTCM), to get the desired shape and size of composite samples. Initially, 30 g of NBR was thoroughly mixed in two roll mixing mill (TRMM) and Ni_0.5_Zn_0.5_Fe_2_O_4_ powder was added in different filler ratio in wt%, as listed in the Table -I. Different additives were mixed in quantity of parts per hundred (PHR) of rubber for vulcanization and curing of the rubber compound. The additives include sulphur (02 PHR) as vulcanizing agent, mercaptobenzothiazole disulphide (MBTS) (1.5 PHR) as accelerator for curing of rubber-filler compound, ZnO (05 PHR), Steric Acid (02 PHR) as activator to enhance the effect of accelerator and Trimethyl-Di-Quinoline TDQ (01 PHR) as antioxidant for rubber to prevent it from environmental degradation^29^. After homogeneous mixing, the compounds were transferred into 200 mm × 200 mm × 2 mm (L × B × D) size mould and hydraulically pressed at 150 °C with 30 Ton pressure for 30 minutes. The mould was then allowed to cool down to the ambient temperature and rubber sheet was collected for MW characterization. The loading of ferrite powder (>80 wt%) in NBR matrix was not possible due to the enhanced brittleness, making difficult to handle the composite materials.

X-ray diffraction measurements (Philips X’Pert Pro) were carried out with CuK_α_ (λ = 1.514 A°) radiation as incident X-ray in 20° to 80° 2θ range, to identify the Ni_1−x_Zn_x_Fe_2_O_4_ crystallographic phase. Infrared (IR) spectroscopic studies were carried out using FTIR system (Model: Bruker Vertex 70 V). Shape and morphology of Ni_1−x_Zn_x_Fe_2_O_4_ powders were analyzed using Oxford EVO5 Scanning Electron Microscope (SEM) system. The room temperature magnetic hysteresis studies (M-H plots) were carried out using Vibrating Sample Magnetometer (VSM) Model ADE EV-5 for −2kOe to +2 kOe magnetic field. Electromagnetic parameters i.e. complex permittivity and permeability values were measured in 2–12.4 GHz MW frequency bands using Vector Network Analyzer model ZVM from Rohde and Schwarz for both pristine Ni_1−x_Zn_x_Fe_2_O_4_ ferrite powders and Ni_1−x_Zn_x_Fe_2_O_4_/NBR composite samples loaded with different concentrations of ferrite powder.

## Additional Information

**How to cite this article**: Saini, L. *et al*. Tunable Twin Matching Frequency (*f_m2_*/*f_m2_*) Behavior of Ni_1−x_Zn_x_Fe_2_O_4_/NBR Composites over 2–12.4 GHz: A Strategic Material System for Stealth Applications. *Sci. Rep.* **7**, 44457; doi: 10.1038/srep44457 (2017).

**Publisher's note:** Springer Nature remains neutral with regard to jurisdictional claims in published maps and institutional affiliations.

## Figures and Tables

**Table 1:** Compositions of MW Rubber Absorbers.

S. No.	Sample Code	Quantity of NBR (g)	Quaninty of Ni_0.5_Zn_0.5_Fe_2_O_4_ firrite in compound (wt%)	Quaninty of ferrite power (g)	Enhancement in filler loading as compared to sample FMAR50 (X)
1	FMAR50	30	50	30	1.00
2	FMAR60	30	60	45	1.50
3	FMAR70	30	70	70	2.33
4	FMAR80	30	80	120	4.00
